# Transcatheter aortic valve replacement with the 34 mm Medtronic Evolut valve: Early results of single institution experience

**DOI:** 10.1007/s12471-018-1122-4

**Published:** 2018-06-25

**Authors:** G. D’Ancona, M. Dißmann, H. Heinze, D. Zohlnhöfer-Momm, H. Ince, S. Kische

**Affiliations:** 1 Department of Cardiology, Vivantes Klinikum im Friedrichshain und Am Urban, Berlin, Germany; 2 Department of Cardiology, Vivantes Humboldt-Klinikum, Berlin, Germany; 3 Department of Cardiology, Vivantes Klinikum Neukölln, Berlin, Germany; 4 Department of Cardiology, Vivantes Wenckebach-Klinikum, Berlin, Germany; 5 Rostock University Medical Center, Rostock, Germany

**Keywords:** Transcatheter, Prosthesis, Aorta, Valve, Large

## Abstract

**Objectives:**

To report our experience with the recently introduced 34 mm Evolut transcatheter aortic valve replacement (TAVR) prosthesis.

**Background:**

A larger TAVR prosthesis has become available for the treatment of aortic stenosis (AVS) in larger native aortic annuli (up to 30 mm). Outcomes with this new device are still unreported.

**Results:**

The first 25 transfemoral TAVRs performed by our team with the self-expandable 34 mm Evolut are presented. The majority of patients were male (84%) with a mean age of 81.3 ± 5.6 years, a median logistic euro-SCORE of 14.7 (5.4-61.0), and a computed tomography measured mean perimeter-derived aortic annulus diameter of 27.1 ± 1.4 mm (min. 25.0–max. 31.2 mm). We implanted one 34 mm Evolut in all patients. Median operative time and radiation time were 68.5 and 12.4 min respectively. To optimise final valve position and haemodynamic performance, at least one complete re-sheathing and re-positioning of the same valve was reported in 33.2%. New permanent pacemaker implantation (PPMI) was necessary in 28.5%. At Receiver Operating Characteristic (ROC) analysis, a minimal diameter of the left ventricular outflow tract <21.9 mm was a significant predictor for PPMI (specificity 82%; sensitivity 83%; *p* = 0.005; Area Under the Curve (AUC) = 0.9). Length of stay in hospital was 9.2 ± 5.8 days and no in-hospital death was reported. At discharge, grade 1 + para-valvular regurgitation was present in 32%, and no regurgitation in the remaining patients. Device success and early safety were 100% and 92% respectively.

**Conclusions:**

TAVR with the 34 mm Evolut prosthesis has shown satisfactory acute outcomes. Although results are consistent with those observed with smaller Evolut prostheses, a trend for a higher PPMI rate has been noticed and could derive from a higher oversizing rate.

## Introduction

The acute safety and clinical performance of the CoreValve Evolut R (Medtronic, Inc., Minneapolis, Minnesota) transcatheter aortic valve replacement system, in patients with severe symptomatic aortic stenosis, have been confirmed in the Medtronic CoreValve Evolut R CE Mark Clinical Study [1] and, subsequently, in the Evolut R U.S. Study [2].

As of February 2017, a larger size Evolut prosthesis has become available in Europe for the treatment of a wider aortic annulus range up to 30 mm. Aim of the present report is to present our experience with the recently introduced 34 mm Evolut transcatheter aortic valve replacement prosthesis.

## Materials and methods

Detailed design characteristics of the Evolut system have already been presented [1, 2]. The system includes a prosthesis (Evolut R) and a delivery system (EnVeo R) with an InLine sheath. The valve and sealing skirt are made of porcine pericardium that has been sutured in a supra-annular position on a compressible and self-expandable nitinol frame. All sizes (23 to 34 mm) have the same overall design and release system, including the possibility to fully reposition and recapture the valve before final complete release. For the 34 mm model, the delivery system has a 16 French equivalent capsule and an InLine Sheath. According to the manufacturer, the 34 mm Evolut is designed to treat aortic annuli with diameters ranging from 26 to 30 mm (perimeter 81.7–94.2 mm).

All patients included in the present series were treated by the same team from February 2017 to July 2017. All patients underwent preoperative multi-slice electrocardiogram-gated cardiac computed tomography (CT) and the 3mensio (3mensio Medical Imaging BV, Bilthoven, NL) image reconstruction programme was used to measure the aortic unit including aortic annulus, aortic root, and left ventricular outflow tract. The aspect of the aortic valve and the presence and distribution of aortic valve calcification were assessed on double oblique transverse reconstructions. Data were prospectively collected, categorised according to the Valve Academic Research Consortium (VARC) criteria [3], and analysed. Early safety was defined as freedom at 30 days from all-cause mortality, all stroke (disabling and non-disabling), life-threatening bleeding events, acute kidney injury—Stage 2 or 3 (including renal replacement therapy), coronary artery obstruction requiring intervention, major vascular complication, and valve-related dysfunction requiring repeat procedure.

All patients had signed an informed consent to data collection and handling for clinical and research purposes. The study was purely observational in nature and for this reason submission to the local scientific/ethical committee was waved.

Data are presented as absolute numbers, percentages, mean ± standard deviation for normally distributed variables and median with minimum and maximum values for variables with non-normal distribution. When sub-group analysis was performed, differences were investigated by means of paired Student *t*-test, Wilcoxon signed-rank test, χ‑square, and Fischer-exact test, whenever appropriate. A *p*-value < 0.05 was considered as significant. The statistical calculations were run using the SPSS 11.0 software.

## Results

During the study period, we treated 73 patients with the Evolut system for severe aortic stenosis. Of these, 25 (34%) were selected for the 34 mm size. At the time of completion of the present manuscript we have treated 8 additional patients with the 34 mm Evolut. Their data are still being collected and are not included in this study manuscript.

The preoperative clinical and cardiac CT data of the first 25 patients treated with Evolut 34 mm are summarised in Tab. 1. The majority of patients were male (84%) with a mean age of 81.3 ± 5.6 years, a median logistic euro-SCORE of 14.7 (5.4–61.0), and a CT-measured mean perimeter-derived aortic annulus diameter of 27.1 ± 1.4 mm (25.0–31.2 mm).

**Table 1:** Demographic and clinical/anatomical data in 25 patients undergoing TAVR with the 34 mm Evolut valve

	*N* = 25
Age (years)	81.3 ± 5.6
Female gender	4 (16%)
Body mass index	27.5 ± 5.4
LVEF %	47.8 ± 13.9
Logistic Euro-SCORE	5.4–61.0; 14.7
Annulus mean diameter (mm)	27.1 ± 1.4
Annulus min. diameter (mm)	23.8 ± 1.8
Annulus max. diameter (mm)	29.5 ± 1.8
LVOT mean diameter (mm)	26.5 ± 1.0
LVOT min. diameter (mm)	22.7 ± 3.0
LVOT max. diameter (mm)	31.2 ± 2.7
Left-coronary sinus (mm)	35.9 ± 2.5
Right coronary sinus (mm)	34.4 ± 2.3
Non-coronary sinus (mm)	36.2 ± 3.3
^a^ *AV calcification degree*	
Mild^b^	3 (12.5%)
Moderate^b^	13 (54.1%)
Severe^b^	8 (33.3%)

Continuous variables are presented with mean ± standard deviation (normally distributed) or interquartile range (IQR) and median (non-normally distributed)

*AV* aortic valve,* LVEF* left ventricular ejection fraction, *LVOT* left ventricular outflow tract, *TAVR* transcatheter aortic valve replacement

^a^Missing information in 1 patient

^b^Calcification Degree: mild calcification with small isolated spots of calcification, moderate calcification with multiple larger spots of calcification, and severe calcification with extensive calcification of all 3 aortic valve leaflets

Intraoperative and in-hospital outcome data are summarised in Tab. 2. Analgosedation and local anaesthesia were adopted in 56% of the patients. The remaining patients underwent general anaesthesia and endotracheal intubation. The right femoral artery was our preferred access site and in the majority of patients we introduced the prosthesis into the vessel with a sheathless delivery system. In 6 cases (17.6%) we used a 20 French femoral sheath to facilitate trackability of the Evolut 34 mm through the femoral-iliac vessels. In 4 patients (16%) a pre-balloon aortic valvuloplasty was performed (one with 20 mm, two with 22 mm, one with 24 mm balloons). All patients were implanted with one single 34 mm Evolut prosthesis that was released under rapid ventricular pacing in 84% of the cases. To optimise final valve position and haemodynamic performance, at least one complete re-sheathing and re-positioning of the same valve was required in 8 patients (32%; one patient required two re-sheathing procedures). No prosthesis post-balloon dilatation was performed.

**Table 2:** Perioperative data in 25 patients undergoing TAVR with the 34 mm Evolut prosthesis

	*N* = 25
Operative time (min.)	25–190; 68.5
Radiation time (min.)	2–38; 12.4
Pre-/post-balloon dilatation	4 (16%)/0
Valve full retrieval	0
*Angiography measurements*	
Valve in-flow (mm)	27.1 ± 3.3
Implantation depth LCS (mm)	8.0 ± 3.3
Implantation depth NCS (mm)	5.2 ± 2.5
*Discharge echocardiography*	
Aortic valve area (cm^2^)	1.8 ± 0.1
Mean gradient (mm Hg)	7.0 ± 3.0
Paravalvular leak (0-1-2)	17 (68.0%)–8 (32.0%)–0
*Cumulative data*	
In-hospital death	0
Device success	25 (100%)
Early safety	23 (92%)

Continuous variables are presented with mean ± standard deviation (normally distributed) or interquartile range (IQR) and median (non-normally distributed)

*LCS* left coronary sinus, *NCS* non-coronary sinus, *TAVR* transcatheter aortic valve replacement*, Device Success* Absence of procedural mortality and correct positioning of a single prosthetic heart valve into the proper anatomical location and intended performance of the prosthetic heart valve (no prosthesis-patient mismatch and mean aortic valve gradient < 20 mm Hg or peak velocity < 3 m/s, and no moderate or severe prosthetic valve regurgitation), *Early safety* freedom at 30 days from all-cause mortality, all stroke (disabling and non-disabling), life-threatening bleeding events, acute kidney injury—Stage 2 or 3 (including renal replacement therapy), coronary artery obstruction requiring intervention, major vascular complication, and valve-related dysfunction requiring repeat procedure

Three patients (12%) required surgical repair of the femoral artery. Final prosthetic in-flow size and prosthetic implantation depth measurements within the non-coronary sinus and right coronary sinus were routinely taken from the last contrasted angiography in the valve release projection (Tab. 2). Median operative time and radiation time were 68.5 and 12.4 min respectively. New permanent pacemaker implantation permanent pacemaker implantation was necessary in 6 of the 21 patients (28.5%) who did not have a permanent pacemaker implantation before transcatheter aortic valve replacement. Univariate analysis showed that patients requiring post-procedural permanent pacemaker implantation had a significantly smaller left ventricular outflow tract minimal diameter (permanent pacemaker implantation 20.3 ± 1.6 mm versus no permanent pacemaker implantation 24.0 ± 3.1 mm; *p* = 0.01) (Tab. 3). At Receiver Operating Characteristic (ROC) analysis, a left ventricular outflow tract minimal diameter cut-off of 21.9 mm had a significant prediction for permanent pacemaker implantation with a specificity of 82% and sensitivity of 83% (*p* = 0.005; Area Under the Curve = 0.9).

**Table 3:** Demographic, clinical/anatomical, and perioperative data in 21 patients requiring and not requiring post-procedural PPMI

	With PPMI (6)	Without PPMI (15)	*p*-value
Age (years)	81.1 ± 5.0	81.0 ± 6.3	0.9
Female gender	0	3 (20.0%)	0.2
Body mass index	26.8 ± 2.8	27.4 ± 5.9	0.7
LVEF%	52.8 ± 6.6	45.7 ± 17.0	0.3
Atrial fibrillation	3 (50.0%)	4 (26.7%)	0.2
A-V block, first degree	1 (16.7%)	1 (6.7%)	0.5
Right bundle branch block	1 (16.7%)	0	0.07
Left bundle branch block	1 (16.7%)	0	0.07
Logistic Euro-SCORE	9.5 ± 4.4	22.1 ± 15.6	0.01
Annulus mean diameter (mm)	26.6 ± 0.5	27.6 ± 1.6	0.2
Annulus min. diameter (mm)	22.9 ± 1.4	24.5 ± 1.9	0.08
Annulus max. diameter (mm)	29.1 ± 1.0	30.0 ± 2.0	0.3
LVOT mean diameter (mm)	25.8 ± 0.9	27.2 ± 0.6	0.03
LVOT min diameter (mm)	20.3 ± 1.6	24.0 ± 3.1	0.01
LVOT max diameter (mm)	30.2 ± 1.1	32.3 ± 3.0	0.1
Left coronary sinus (mm)	36.1 ± 1.3	35.6 ± 3.0	0.7
Right coronary sinus (mm)	34.9 ± 2.1	34.1 ± 2.4	0.4
Non-coronary sinus (mm)	36.0 ± 2.2	36.4 ± 3.9	0.8
^a^ *AV calcification degree*			
Mild	0	2 (14.2%)	0.7
Moderate	4 (66.7%)	8 (57.1%)	
Severe	2 (33.3%)	4 (28.5%)	
Implantation depth LCS (mm)	9.0 ± 2.6	7.4 ± 3.7	0.2
Implantation depth NCS (mm)	6.0 ± 2.2	5.1 ± 2.9	0.5
Length of stay in hospital (days)	11.5 ± 4.0	8.4 ± 5.0	0.2

*AV* aortic valve, *A-V block* atrioventricular block, *LCS* left coronary sinus, *LVEF* left ventricular ejection fraction, *LVOT* left ventricular outflow tract, *NCS* non-coronary sinus, *PPMI* permanent pacemaker implantation

^a^Missing information in 1 patient

^b^Calcification Degree: mild calcification with small isolated spots of calcification, moderate calcification with multiple larger spots of calcification, and severe calcification with extensive calcification of all 3 aortic valve leaflets

Continuous variables are presented with mean ± standard deviation (normally distributed) or interquartile range (IQR) and median (non-normally distributed)

Length of stay in hospital was 9.2 ± 5.8 days and no in-hospital death was reported.

Device success and early safety were 100% and 92% respectively. Two patients (8%) experienced stage 2 acute kidney injuries and one patient (4%) post-operative delirium. At discharge, grade 1+ para-valvular regurgitation was present in 32% and no regurgitation in the remaining patients. Average mean trans-prosthetic gradient was 7.0 ± 3.0 mm Hg (Tab. 2).

## Discussion

At present the Evolut R 34 mm is designed to address the widest aortic annulus range of any other commercially available transcatheter aortic valve replacement prosthesis. The manufacturing company suggests some anatomical reference ranges, such as an aortic valve annulus perimeter-derived diameter of 26–30 mm, an annular perimeter of 81.7–94.2 mm, a mean sinus Valsalvae diameter of at least 31 mm, and a mean sinus height of at least 16 mm. Our report shows that the valve achieves immediate clinical and haemodynamic results that are consistent with those observed with the smaller models of the Evolut R system [1, 2] when we follow these anatomical guidelines. The specific features of controlled and accurate deployment allow for precise positioning to achieve the best haemodynamic performance even in larger annuli. When we look more specifically at our findings, we see that we were also able to treat patients with an annular perimeter-derived diameter up to 31 mm, maintaining a limited rate of para-valvular leaks.

The mechanism of re-sheathing (re-capture) can be adopted, even multiple times, always while maintaining adequate haemodynamics, without damaging the re-sheathing mechanism and the prosthetic structure. In this context, we found the re-capturing feature particularly helpful in patients with a larger annular anatomy and/or a lower degree of aortic valve calcification. According to our findings, we experienced that at least a third of patients will benefit from a complete valve re-sheathing and optimised repositioning.

Although the overall trackability of the device is excellent, even within very tortuous anatomy, some comments should be given about its trackability within the femoral-iliac vasculature, particularly at the entry point within the vessels. According to the manufacturing company, the built-in InLine sheath allows for the complete system to be inserted directly without the need for a separate access sheath. We have learned that in some cases pre-dilatation of the femoral access with a 16 French sheath or insertion of the Evolut via a 20 French sheath may facilitate system advancement within the first tract of the femoral vessels and in this way minimise vascular trauma. This is particularly helpful when dealing with patients with abundant subcutaneous tissue and obese habitus.

Finally, our reported permanent pacemaker implantation rate of 28% with the Evolut 34 mm seems higher than the rates presented in the Evolut R CE Mark Clinical Study (11.7%) [1] and in the Evolut R U.S. Study (16.4%) [2], and higher than the rate recently proposed in a more contemporary cohort of patients treated with the Medtronic Core valve (18.2%) [4].

In our personal contemporary experience with the smaller Evolut prostheses we have observed a permanent pacemaker implantation rate in the 20% range.

There are multiple clinical and anatomical (existing atrioventricular and intraventricular conduction delays and/or atrial fibrillation, aortic valve annulus calcification degree) and operative variables (prosthesis type and implantation depth) that may impact upon the permanent pacemaker implantation rate after transcatheter aortic valve replacement [4]. In light of the limited sample size we cannot draw evidence-based conclusions, even though some of those variables are partly confirmed in our presented experience with the 34 mm Evolut.

**Fig. 1:**
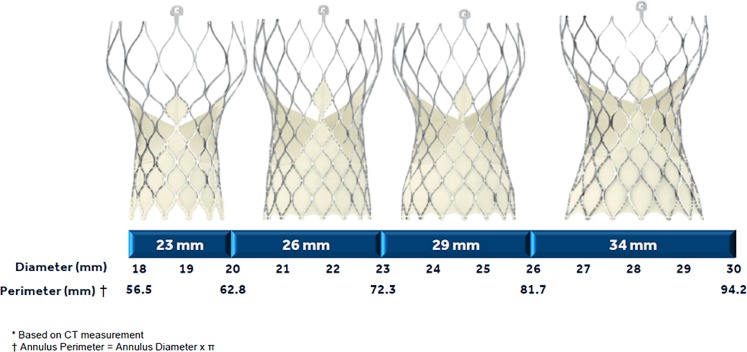
CoreValve Evolut R size selection according to range of native aortic annulus diameters/perimeters (courtesy of Medtronic, Inc., Minneapolis, Minnesota)

In any case, there is overwhelming evidence that prosthesis implantation depth during the peri-procedural phase plays an important independent role in post-procedural permanent pacemaker implantation rates [4]. Implantation depth can gain further importance when adopting large size prostheses. Therefore, the 34 mm Evolut may increase the overall oversize rate within the native aortic annulus and left ventricular outflow tract because it has a wider range of annular diameter applicability (annular perimeter ranging from 81.7–94.2 mm; 12.5 mm range) than the smaller models (6.3, 9.4, 9.4 mm ranges for the 23, 26, 29 mm Evolut) (Fig. 1). Excessive valve oversizing may eventually increase the possibility of atrioventricular node trauma. Interestingly, in the present limited experience with the Evolut 34 mm we have observed that permanent pacemaker implantation was significantly more common in patients with significantly smaller left ventricular outflow tract diameters. In these patients, valve oversizing may have played an important role in compression and oedema in the vicinity of the conduction system, within the left ventricular outflow tract. What emerges from our limited analysis is that in the selection of prosthesis size, left ventricular outflow tract diameter should be considered. To reduce the chances for permanent pacemaker implantation, the operator may choose a smaller size prosthesis in patients with aortic annulus measurements that are borderline for either a 29 or 34 mm Evolut when the minimal diameter measurements of left ventricular outflow tract are below a certain value (21.9 mm in our experience).

## Limitations

We should restate that the aim of the present study was not to compare patients according to their Evolut prosthesis size. Furthermore, identifying permanent pacemaker implantation determinants is beyond our tools because the presented sample with 34 mm prostheses is limited. Findings presented in this study have to be interpreted with caution and as hypotheses generating.

## Conclusion

To the best of our knowledge this is the first ever published complete report about the self-expandable 34 mm Evolut transcatheter aortic valve replacement prosthesis, apart from a previous poster presentation [5]. Data represent the initial experience with this new device in a real-world single institution. When adopted within the premises of teams that already have a wide experience with transcatheter aortic valve replacement using both the Evolut and other transcatheter aortic valve replacement devices, the 34 mm Evolut system has shown satisfactory acute clinical and haemodynamic results in the treatment of patients with severe aortic stenosis.

In this initial and limited series, requirement for post-procedural permanent pacemaker implantation after transcatheter aortic valve replacement with the 34 mm Evolut seems slightly higher than that observed when using smaller Evolut prostheses. This finding needs further investigation and may be related to an increase in the 34 mm valve oversize rate, particularly within the native left ventricular outflow tract.
